# Evaluating transfer prediction using machine learning for skill acquisition study under various practice conditions

**DOI:** 10.3389/fpsyg.2022.961435

**Published:** 2023-01-19

**Authors:** Anna Aniszewska-Stȩpień, Romain Hérault, Guillaume Hacques, Ludovic Seifert, Gilles Gasso

**Affiliations:** ^1^LITIS EA4108, INSA Rouen Normandy, Normandy University, Saint-Etienne-du-Rouvray, France; ^2^CETAPS EA3832, Faculty of Sport Sciences, University of Rouen Normandy, Normandy University, Mont-Saint-Aignan, France; ^3^Institut Universitaire de France, Paris, France

**Keywords:** learning dynamics, variable practice, constant practice, transfer skill, predictive model, linear regression, feature selection

## Abstract

Recent research highlighted the interest in 1) investigating the effect of variable practice on the dynamics of learning and 2) modeling the dynamics of motor skill learning to enhance understanding of individual pathways learners. Such modeling has not been suitable for predicting future performance, both in terms of retention and transfer to new tasks. The present study attempted to quantify, by means of a machine learning algorithm, the prediction of skill transfer for three practice conditions in a climbing task: constant practice (without any modifications applied during learning), imposed variable practice (with graded contextual modifications, i.e., the variants of the climbing route), and self-controlled variable practice (participants were given some control over their variant practice schedule). The proposed pipeline allowed us to measure the fitness of the test to the dataset, i.e., the ability of the dataset to be predictive of the skill transfer test. Behavioral data are difficult to model with statistical learning and tend to be 1) scarce (too modest data sample in comparison with the machine learning standards) and 2) flawed (data tend to contain voids in measurements). Despite these adversities, we were nevertheless able to develop a machine learning pipeline for behavioral data. The main findings demonstrate that the level of learning transfer varies, according to the type of practice that the dynamics pertain: we found that the self-controlled condition is more predictive of generalization ability in learners than the constant condition.

## 1. Introduction

In the proposed approach, we have raised the still open question about the conditions of practice that are most beneficial for motor learning—is the variability of the task or goal during practice advantageous or detrimental for the learning effect? Which type of practice, constant or variable, is more profitable in terms of performance and skill transfer to novel conditions? We claim to answer these questions through machine learning algorithms. Machine learning would help explain the learning process by modeling and predicting phenomena such as the transition between movement patterns, the flexibility of a given movement pattern (e.g., range of motion), the rate and dynamics of learning, and the skill and learning transfer. In our study, we used a predictive model. Prediction is a predominant manner to evaluate the rate and dynamics of learning through a statistical approach, but it can likewise evaluate the post-practice effect known as retention and transfer (e.g., skill and learning transfer). The next subsection outlines the key features of skill acquisition in humans, to explain how machine learning can contribute to a deeper understanding of the learning and transfer process.

### 1.1. Learning dynamics

Within the ecological dynamics framework (Button et al., 2020), learning is defined as the entire reorganization of the perceptual-motor intrinsic dynamics of the learner (i.e., behavioral repertoire). Such behavioral reorganization, in particular, the nature of the change (shift vs. bifurcation), involves the interplay of dynamic processes that are cooperation and competition between the intrinsic dynamics and the behavioral information (e.g., task goal) (Zanone and Kelso, 1992; Button et al., 2020). In other words, learning some tasks more easily than others depends on the extent to which behavioral information cooperates or competes with the intrinsic dynamics. If the pattern to be learned is near one of the intrinsically stable patterns, cooperative processes predominate and the learners quickly enhance their performance without exhibiting a bifurcation between patterns, but a shift and refinement of existing patterns. On the contrary, if the behavior required by the task is far from the initial pattern contained in the intrinsic dynamics, competition processes would lead to a drop in the stability of the existing pattern, and a bifurcation or at least an intermittent regime between the existing new patterns will occur (Zanone and Kelso, 1992; Button et al., 2020). To sum up, learning is a dynamic and continuous process, during which learners are always navigating between competition and cooperation processes, according to their improvement, which is probed and updated continuously through the mutual and reciprocal coupling between perception and action (Button et al., 2020). When the task goal is set (and thus the multiple movement possibilities are reduced to a certain subset) and with practice, performance stabilizes as the learner discovers and exploits effective movement solutions that fit the task dynamics (Davids et al., 2012). In this way, from the constant interchange of perception and action, the individual's learning model is attuned by a continuous flux of information that reflects coordination dynamics (Kelso and Fuchs, 2016). Likewise, the number of coordination patterns (Edelman and Gally, 2001; Price and Friston, 2002) is reduced to a low-dimensional structure (Liu et al., 2003). The individual learner's path to acquire his stable behavior, as manifested in the dynamics of performance, is believed to be modeled by a monotonic function and the most prevalent type of this function studied in the literature is the exponential function. The exponential function can at the same time be designed to grasp the essence of learning dynamics. We comment more extensively on our choice of modeling function curve in Section 2. Our primary goal was to model the individual learning dynamics to consider the inter-individual variability that could occur during learning and, more importantly, that could occur depending on the nature of the practice (constant vs. different forms of variable practice, as described in the next section).

### 1.2. Induced variable practice in learning protocol

Assuming that multiple coordination patterns (expressing the exploitation of degeneracy of perceptual-motor systems; see Seifert et al., 2016 for further details) can emerge within and between learners for a given task goal, a selection among those patterns or an intermittence between these patterns would reflect how learners adapt to the task goal, especially when variable practice is induced. Despite the pioneering work of Shea and Morgan (1979) who long ago demonstrated that transfer was greater in groups with random acquisition than in groups with a blocked acquisition, there is an ongoing debate about how inducing variability during practice facilitates learning in the terms of finding the spectrum of solutions that help the system generalize adapted skills. Indeed, most studies demonstrate that increasing variability slows down learning but has a beneficial effect on generalization (Raviv et al., 2022). Moreover, a similar effect has been found in various domains, including motor learning and machine learning. The most interesting questions, thus, concern the impact of the amount of variability, its mode of application, and the quantifiable effect it brings to the generalization level.

Two types of externally induced variability can be distinguished (Ranganathan and Newell, 2013). The unstructured variations, usually applied with random rigor, employ the modification of multiple variables at the same time to perturb the proficiency of the performer (Frank et al., 2008). They are contrasted with the structured variability (Braun et al., 2009), which involves only one variable so by observing the change they engender in the particular learning outcome, we are allowed to explicitly match the operational variable with the shift in performance (Newell et al., 1991; Pacheco et al., 2019). When contrasting these two approaches, the results from Hossner et al. (2016) indicated that a learning protocol, in which some variables are varied and others are not changed from the previous trial, was more effective in post and retention tests than a learning protocol involving variations in all variables. This result suggests that more structured variations could more effectively guide learning.

Moreover, recent results demonstrated that practice conditions leading to excessive exploration of movement solutions can be detrimental to learning (Sidarta et al., 2022). In fact, newly discovered behavioral solutions require exploitation during practice to stabilize them in the learners' repertoire (Hossner et al., 2016; Komar et al., 2019). Since the ratio between exploration and exploitation of coordination patterns differs among individual learners, giving them some control over their practice conditions could be more respectful of the individual learning dynamics. Such practice conditions have been tested and have revealed promising results suggesting that participants applied the given control to optimize their practice schedule to their needs (Liu et al., 2012). Indeed, giving self-control over the practice schedule to learners would be consistent with the key property of emergence observed in a dynamic system, which is expressed by functional adaptive behavior in learning dynamics. In this regard, we will test this claim in our approach.

### 1.3. Transfer test

To understand how variable practice (and its various forms, e.g., teacher-led practice vs. self-controlled by learners) could be beneficial to the development of functional adaptive behaviors, it is needed to overcome the traditional retention test used to assess learning and to evaluate as well the learning generalization by measuring the skill transfer to the new context. The skill level acquired during practice is in this case followed by a variant condition (in the case of a climbing task—a new route) for which performance is assessed. In comparison with another post-practice test, the retention test, which is the measure of the stability of the acquired skill level over time (or the inverse of forgetting), the formal definition of skill transfer emphasizes the degree of similarity (or difference) of the original, practiced condition (Pacheco and Newell, 2018b), with the transfer condition (Ranganathan et al., 2014). In our approach, we predict the value of performance transfer (climbing fluency level), based on the spatial and temporal performance dynamics during practice (reduced to the form of fitting function parameters). The accuracy of this prediction is subsequently used, comparatively, to judge whether the type of practice was predictive of generalization, that is, whether learners developed adaptive behavior to produce the skill transfer effect.

A similar approach (but based on the raw biomechanical variables rather than on performance metrics, as was done in our case) was used in Pacheco and Newell (2018b) to trace variability in constantly practicing individuals. Our approach, however, extends the previous analysis and compares the constant practice result with the variable practice conditions that we consider crucial to judge the stability of the learners' dynamics under both practice conditions (Pacheco and Newell, 2018a).

### 1.4. The rationale of the approach

For all the aforementioned reasons, repetitive practice on an ongoing basis (constant practice under unchanged conditions) seems insufficient for gaining knowledge about transfer prediction in motor skill acquisition. Even if the variability of some degree is always present in the learner–environment system, it would probably be beneficial to introduce the variable practice, which should adapt tasks design to the needs and motivation of the learner along the learning process. It is likely that in the absence of confronting the effects of constant and variable practice, we would not be able to capture the full range of learning dynamics. Therefore, the selection of appropriate assessment (in terms of measuring the level of variability), becomes crucial (Section 4) both during the implementation of practice variants (training session) and at the end of the learning process (testing session, in our case, transfer trial).

In our study, we undertook the challenging task of evaluating measures of learning used in behavioral neuroscience applied to human movement science, under assumptions that allow for simple application and interpretation. Anchored within the framework of ecological dynamics (Button et al., 2020), our main objective was to assess how learners functionally adapt their behavior during constant and variable practice in a learning protocol and then to predict how different forms of variable practice could help to more efficiently transfer their skills to a new situation. Thus, in light of this framework, we questioned how the shape of the dynamics (steaming from a given practice) is predictive of reaching a high level of transfer. The presented analysis is based on the climbing training of three groups of participants, who followed either training on only one climbing route (constant practice), variants of that route (variable practice), or on variants of the route but with the opportunity to practice on the same routes for several sessions (self-controlled practice), for which the skill transfer test (on a new climbing route) was used for validation. For each route, the performance scores of the participants, efficiency (understood as fluency of movement or smoothness, detailed in Supplementary Section 2.1) were calculated, which gave us general access to the participants' dynamics (i.e., time series of fluency indicators). Climbing fluency (as exhaustively exposed in the study by Seifert et al., 2014) proved to be a measure that in different aspects (spatial and temporal, depending on the indicator in use) corresponds to the learners' skillfulness in the climbing task. We assumed that both constant practice (repetitive training on a single route) and variable practice (with route variants introduced) might be effectively evaluated by the test of transfer of acquired skill to a new context. In our study, this measure in each practice condition (constant and variable) was matched with methods used in the machine learning (Belloni and Chernozhukov, 2013) to predict the training outcome (total effect of training) based on input (learning dynamics reflected in ongoing training scores throughout the climbing training protocol).

The rationale for employing the statistical method to trace the effectiveness of transfer is rooted in the presumed attribute of this test to track the ability of the climber to generalize to new climbing routes. Indeed, during climbing, especially outdoors, climbers are always faced with new environments and new routes. Obviously, they may train on the same route if they do not succeed or they wish to improve their fluency (which justifies the distinction between imposed variable practice and self-controlled variable practice groups). However, the essence of climbing is to be able to climb “on-sight” a new route (i.e., without any prior knowledge of the route). Furthermore, by applying the reduction of the learning signal (the learner performance history) measured by four metrics (entropy, jerk, immobility ratio, and climbing duration, detailed in Section 2), to the parameters of an exponential function fitted to it, we were able to significantly reduce the dimensionality of the machine learning prediction problem. Thus, in our analysis, the learning dynamics could be captured by just a few parameters, which facilitates machine learning algorithms that could be applied to compare the transfer test prediction under different conditions.

To summarize, in our study, by means of machine learning predictive algorithms, we attempt to address the problem of learning evaluation with a transfer test applied to different conditions of climbing practice (constant or variable one). The method provided assumes the relation between the relevance of test attribution in human movement science and statistical predictivity understood as the relationship of the output data (transfer test scores) to the input time series (variables of learning dynamics). In further sections, we will describe the methods used in the study (Section 2), brief the results (Section 3), and discuss the results and their interpretation (Section 4).

## 2. Methods

### 2.1. Data collection

#### 2.1.1. Climbing experimental setup

A group of 34 student volunteers from the University of Rouen Normandy (11 female students and 23 male students) were recruited to participate in this study. On average, the participants were 20.3 ± 1.2 years old, 172.3 ± 6.8 cm tall, and 66.4 ± 9.8 kg, and had an arm span of 172.7 ± 8.6 cm. Five participants were left-handed, and the remaining 29 were right-handed. One participant dropped out of the study during the training sessions due to injury.

**Figure 1:**
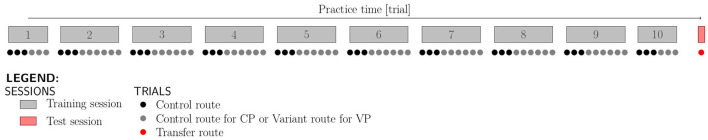
Protocol of data collection for the acquisition of a behavioral signal. The rectangular blocks in the top figure count the sessions. The dots in the bottom figure illustrate the number of trials in the sessions, which was the same for all the groups (CP, VP1, and VP2). The color of the dots distinguishes the variability that was introduced. The black dots represent the route that was identical for all the climbers in all the sessions and which was the *control* route. The gray dots represent either 1) control route in the case of the CP group, or 2) *variant* route in the case of the VP1 and VP2 groups. The last red dot symbolizes the *transfer* route (test route which was different from the control route or variant routes), which is used to assess participants' progress when faced with a novel context.

The participants of the experiment were climbing designed routes on an artificial wall. They were novice climbers following training consisting of 84 trials divided into 10 training sessions that have been performed within 5 weeks with two sessions per week (within 5 working days, excluding the weekend), with at least 1 day of rest between two sessions. The training concluded with one trial on a route, where the participants did not train. This route was called the *transfer route*. The climbing protocol has been thoroughly described in Hacques (2021). The necessary elimination (resignation of one participant and exponential divergence of three participants' data, explained further) resulted in a total of 30 participants being included in the analysis: Nine of them (constituting the constant practice group CP) were following the very same route (called the *control route*) throughout the training. The remaining 21 climbers (constituting the variable practice VP group) were climbing the same route (control route) only on the first three trials of each session, while the remaining trials in a session (three in sessions 1 and 10, and six in sessions 2 through 9) were performed on different routes (called *variant routes*). Nonetheless, all the groups practiced the same amount of trials per session. The subgroup of the climbers who climbed the altered routes (VP) could choose at the end of each session whether they wanted to continue their practice on the same routes or be confronted with a new route (VP2 subgroup). The rest of the VP group (VP1 subgroup) performed the instructor-controlled protocol and were confronted with the new climbing route at each learning session. The protocol is illustrated in Figure 1 and the climbing routes examples in Supplementary Figure S2 in Supplementary Section 2.1. By examining route modifications, we were able to clearly discern the differences in the route designs. The route conversions did not account for the handle shape modifications or the rotation of the handle and focused mainly on their displacement (due to the climbing variability detailed study Hacques et al., 2021).

**Figure 2:**
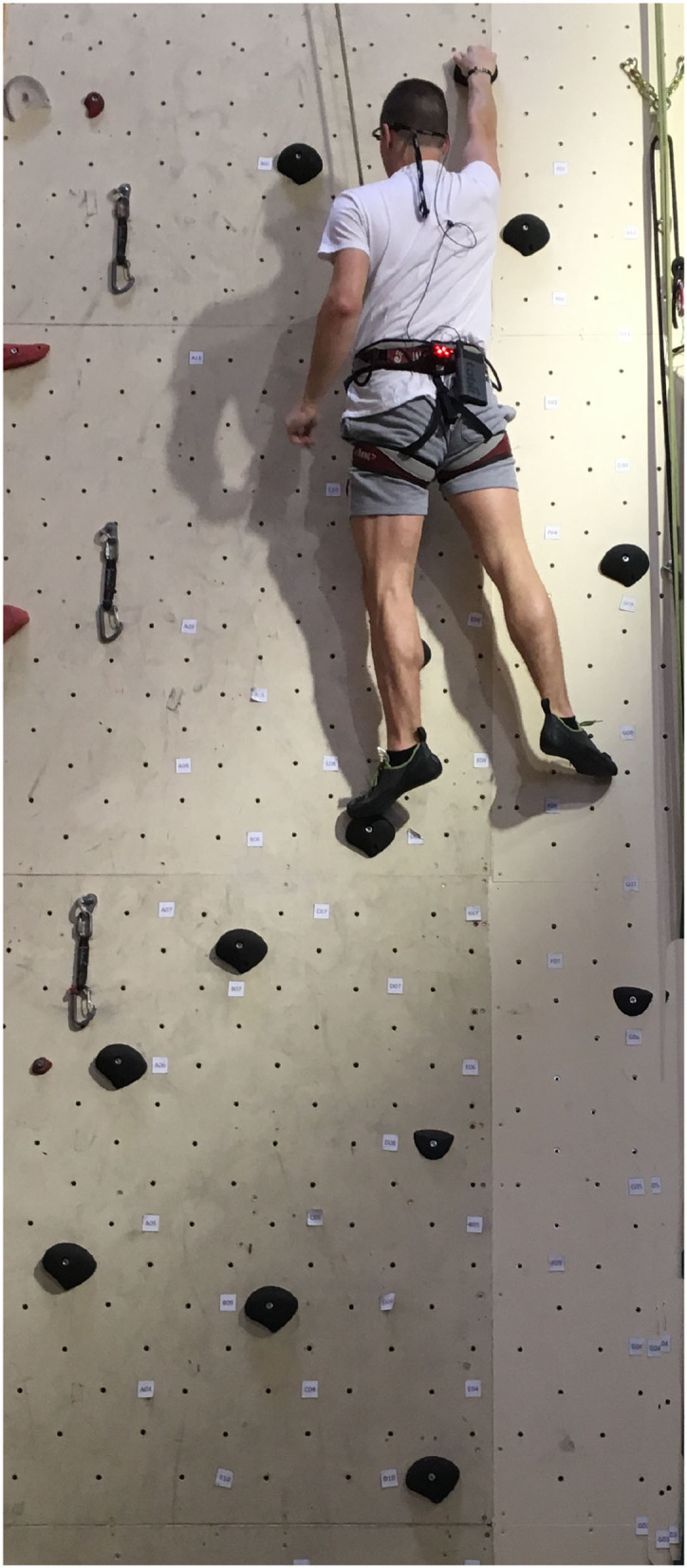
Climbing route setup: the led lamp light to track the trajectory of the climber is attached to the harness along with the IMU. The handholds on the artificial wall are equipped with sensors.

The hip coordinates of the participants were recorded based on the trajectory of a red light provided by a LED lamp attached to the climbing harness. Moreover, participants had a Hikob IMU (Inertial Measurement Unit) placed on their back. The IMU contains an accelerometer, a gyroscope, and a magnetometer (Figure 2). Some of these recordings (position and acceleration) were used to calculate the fluency indicators for each of the 84 training session trials and one post-training transfer route (detailed later in the present section). Before each session opening, climbers were given feedback^1^ about their climbing fluency during the preceding session.

#### 2.1.2. Ethics statement

The protocol followed the guidelines of the Declaration of Helsinki. Procedures were explained to the participants who then gave their written consent to participate.

### 2.2. Modeling the climbing learning signal

#### 2.2.1. The reviewed signals

Three measures of fluency, which were precalculated for the purpose of the study from the climbers' trajectory, were used to assess their performance while climbing each route: geometric index of entropy (GE with units [bits] or [log2]), jerk of the hip acceleration (JE, dimensionless), and the ratio of hip immobility (IM measured in [s]), as in the Supplementary Section 1. All of them are classic measures employed in the evaluation of motor performance in climbing (Cordier et al., 1994; Seifert et al., 2014; Orth et al., 2017) and describe the smoothness of movement during climbing of each route (spatial fluency for GE, spatio-temporal fluency for JE, or purely temporal fluency for IM). In addition, as an auxiliary measure^2^, we used climbing time (CT, measured in [s]).

**Figure 3:**
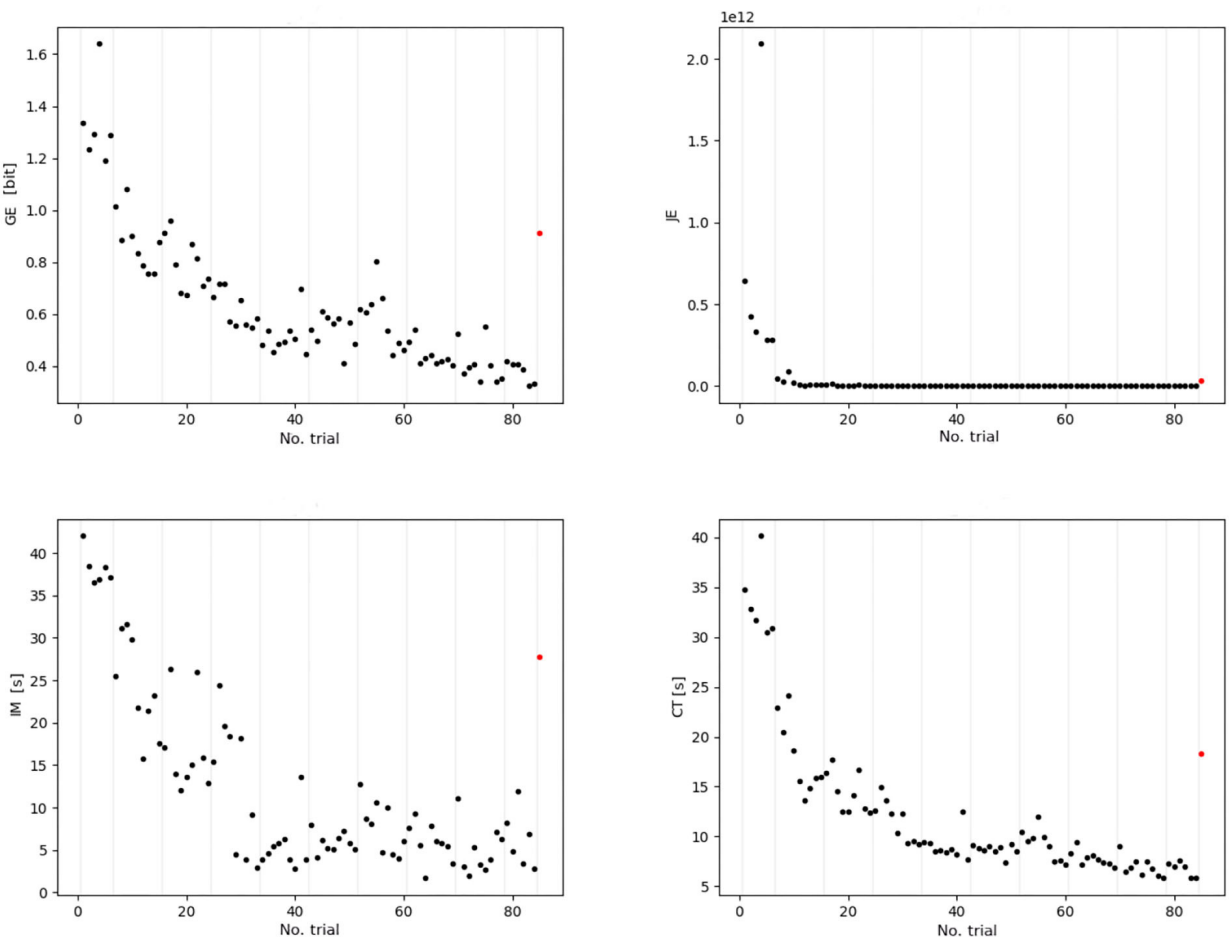
Four metrics (GE, JE, IM, and CT) of one participant of the VP group (ordinate axis indicates raw value scale, i.e., the values prior to standardization). The black dots represent parts of the signal of each indicator that will be preprocessed to build the input features *via* exponential fitting, they correspond to the training routes. The red dot exhibits the target value to be predicted; these target values correspond to the last *transfer* route. The gray vertical lines indicate the session's division. It is worth noting that the transfer route fluency score is of different nature than the practice session scores; thus, it cannot be estimated by extrapolating the exponential regression.

Figure 3 presents the examples of behavioral signals obtained by computing the fluency indicators of each trial for one training participant. The trials' fluency constituted the training session scores and was taken into account as training features in the machine learning algorithm. They shape the *learning curve*, whereas the transfer trial fluency (post-training evaluation score), which appears each time as a single red dot, accounted for the prediction target in the statistical learning. We can observe that the transfer fluency is of a completely different nature than the learning function. Moreover, as the climbing raw fluency values for different indicators demonstrate a large range of discrepancies (from 10^−12^ for jerk to 10^1^ for immobility and duration time), before any processing, we applied standardization of data. It is worth noting, that for the input data, we utilized four metrics (including climbing duration CT), while only three fluency indicators were our prediction outputs (GE, JE, and IM). Thus, we utilized four types of metrics as features and three metrics as targets (Table 1).

**Table 1:** Set of 8 features used for prediction of the 3 targets (*y*^*I*^, *I* = {*GE, JE, IM*}).

**Features (computed on training phase)**								**Targets (transfer phase)**
*a* ^*GE*^	*e* ^*GE*^	*a* ^*JE*^	*e* ^*JE*^	*a* ^*IM*^	*e* ^*IM*^	*a* ^*CT*^	*e* ^*CT*^	*y* ^*JE*^


#### 2.2.2. Signal preprocessing and feature extraction

For the prediction purpose, the main challenge that we face from the machine learning point of view is the large number of features in comparison with the small set of samples we dispose of. We addressed this problem in two possible manners: by fitting the exponential function to the measurements and by a further feature selection step implemented in the first procedure of our prediction algorithm (STEP 1 in Algorithm 1).

**Algorithm 1:**
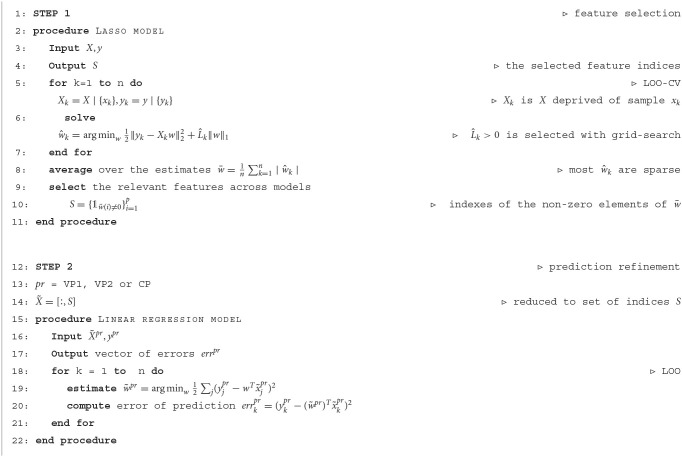
Two-stage model (one indicator case)

**Figure 4:**
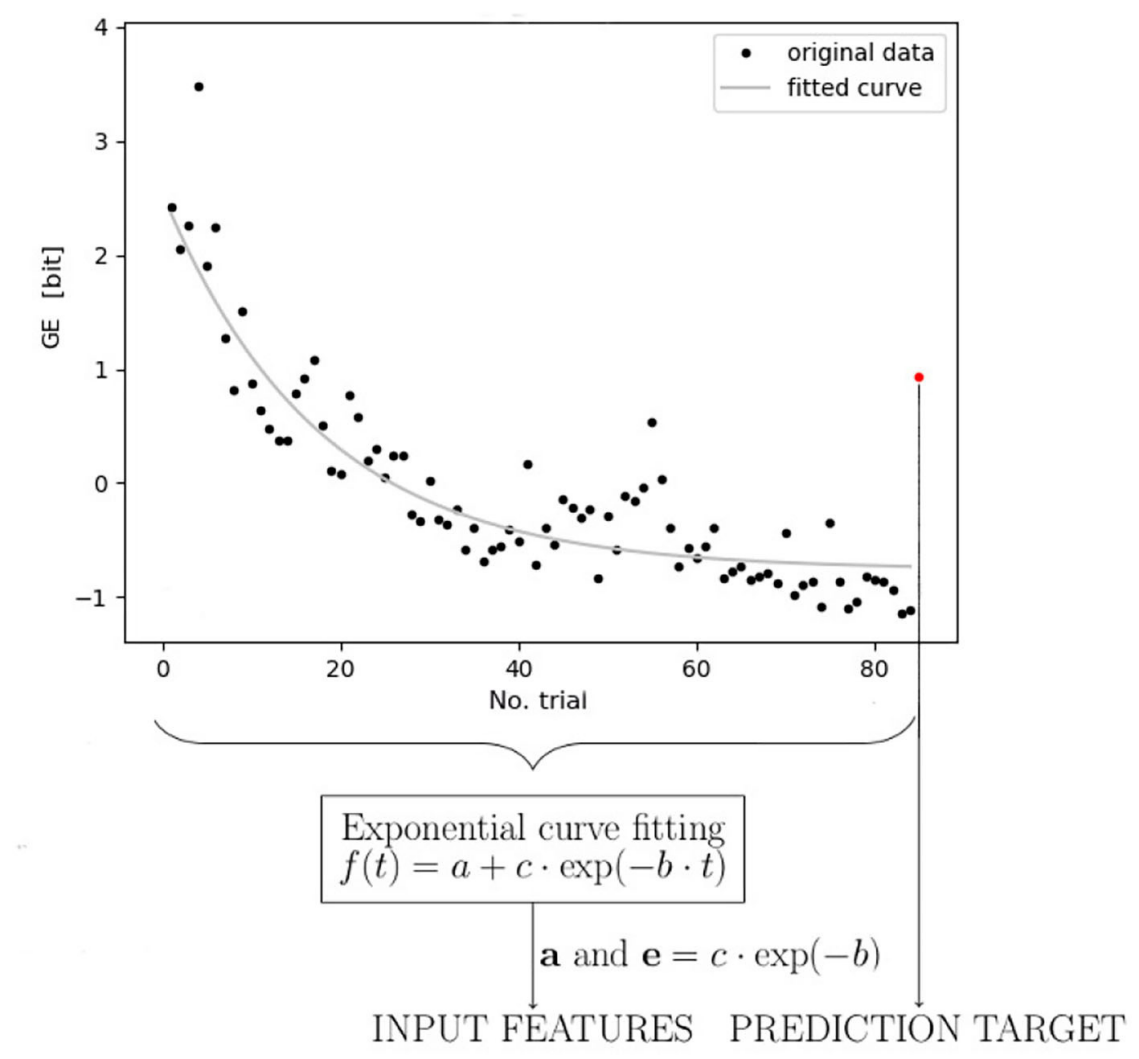
Diagram of the modeling stages. The *first stage*, illustrated as black dots in the figure, depicts fluency calculation for the training data of one participant, and the last red dot symbolizes climbing fluency for the transfer route in the post-test trial. In the *second stage* (learning dynamics modeling), the exponential function is fitted to the training data signal, except for the transfer test value (unmatched). Subsequently, the exponential function parameters of all metrics (entropy, jerk, immobility ratio, and climbing duration) are utilized in the prediction algorithm (*third stage*) as an input set of features, while the transfer value is utilized as an output (target). This stage models the learning generalization. The exemplary entropy data in the graph have been standardized beforehand.

As mentioned in Section 1, the exponential curve has been postulated as a function that reflects the learning progress among the trainees and provides the best fitting to the learning data. In the comparative study (Newell et al., 2009), the authors referred to established training data (data for testing fine visual-motor skills: pursuit rotor task Adams, 1952 and mirror tracing task Snoddy, 1926) and evaluated the power law and exponential models with single- and double-time dynamics. In the rationale, the authors mention (as in Mayer-Kress et al., 2006) the slow dynamical evolution of the learning, which accounts for general memory involvement, was additionally furnished with the second time parameter. This parameter represents the adaptation process within the ongoing session to grasp the fast changes, usually predominant during the rest periods (outside the scope of the measurements, thus their immediate presence remains unregistered). However, even if the slow time dynamics progress is undeniably present in our data (reflecting the stability of the learning curve), patterns that reflect the fast time scale (adaptation) are not possible to be captured in a task that induces high fatigue, such as climbing (thus we dispose of too few trials per session to reflect the fast scale effect). Hence, in our study, we focus on modeling the slow dynamics using a decreasing exponential function (Figure 4).

Exponential curve fitting is intended to summarize the climbing training dynamics in a few features with which we attempted to obtain the lowest number of parameters (features) possible (to apply the sequence of fluency indicators on sessions to the prediction algorithm in further steps). Thus, if we focus on the predominant tendency and reduce the number of parameters involved in the exponential models, we might approximate the dynamics as close as possible with a simple exponent that could be ultimately symbolized with only two terms. In our approach, the first parameter (an additive part *a* present in the formula Equation 1) would refer to the maximum performance of each participant (understood as the inverse of the fluency measure) achieved during training (the asymptotic minimum of the exponential decay, which is an *inverted plateau* and a stable state of the learning dynamics) while the second one (the exponential parameter in Equation 1, i.e., *e* = *c*·exp−*b*) would depict the individual learning rate of each participant. This way, the simplified two-parameter approach satisfies both: motor description exhaustion (using as many parameters as necessary) and data processing convenience (using the least number of features).

The exponential model we employ to reflect climbers dynamics is


(1) $$\begin{array}{c} f\left(t\right)=a+c\cdot \exp\left(-b\cdot t\right) \end{array}$$

With parameters *a*, *b*, and *c* (Figure 4). For each climber and each of the related fluency indicators (GE, JE, and IM), we fitted the exponential model by solving


$$\underset{a,b,c}{\min}\frac{1}{n}\underset{t}{\sum }{\left(a+c\cdot \exp\left(-b\cdot t\right)-I\left(t\right)\right)}^{2}$$

Where *I* denotes the fluency indicator (GE, JE, or IM). Hence, for each climber, the estimated parameters serve as features to predict the outcome of learning progress in the post-training *transfer route* fluency. Specifically, we consider *a* (an additive parameter) and *e* = *c*·exp^−*b*^ (exponential parameter) as features associated with each fluency indicator (Table 1).

Even though the exponential model adopted here is justified for learning curve modeling, we must be cautious since the individual intrinsic dynamics (the inter-participant differences in pre-training dynamics Kostrubiec et al., 2012) and the context of motor activity (e.g., the different sports) may promote different learning functions, as explained in the work (Newell and Liu, 2012).

#### 2.2.3. Sum-up of the input features

For post-training transfer score prediction, we used the parameters of the exponential function fitted to the training performance based on all four metrics (entropy, jerk, immobility, and duration). Therefore, after reducing the parameter number to 2 (an additive, *a*, and an exponential term, *e* = *c*·exp(−*b*)), we used altogether eight features to predict each indicator's transfer value for the datasets that consisted of 9 (CP) and 21 (VP) samples (9 in VP1 and 12 in VP2).

We underline that (to be able to apply linear regression algorithms), in the prediction, as in the exponential fitting procedure, our priority was to reduce the number of features, while not discarding essential information from data. The involved initial specification for our model is detailed in Table 1.

#### 2.2.4. The prediction algorithm

In our approach, we applied the linear model, suspecting a linear relationship between input (the joint exponential parameters of learning dynamics for all the indicators) and output (test transfer fluency). For this aim, as mentioned in Section 1, we evaluated the model predictions on each set of practice separately ( VP1, VP2, or CP).

For the *y*∈ℝ^*n*^, which is the output (transfer tests vector), and *X*∈ℝ^*n*×*p*^, which is our input of the parameters of exponential curve fitted to the fluency indicator history (learning dynamics), where *n* and *p* are, respectively, number of the samples (30) and of the features (8), we can formulate a linear model. In order to apply the least squares linear regression model, a necessary condition is that *p* ≤ *n*. Due to Table 1, it is possible for VP1, VP2, and CP; however, by applying the pre-train selection procedure with Lasso (Belloni and Chernozhukov, 2013) (STEP 1) in advance of linear regression application (STEP 2), we could improve our prediction result (i.e., we are guaranteed not to degrade it). The method for finding the best coefficients (weights) *w* in the linear models (both steps) was leave-one-out cross-validation (LOO-CV) (James et al., 2021). Lasso (Tibshirani, 1996) is known to perform as a model selection method due to its properties of zeroing out the unimportant coefficients in the model and returning the sparsified vector of weights for further processing. A prerequisite of the two-stage approach (Belloni and Chernozhukov, 2013) is that we should expect a sparse model. In Lasso hyper-parameter tunning, the grid search was employed to find the best $\hat{L}$ value (Hastie et al., 2009). We configured the two-stage model as follows in the Algorithm 1 (specified for one indicator).

## 3. Results

### 3.1. Exponential fitting evaluation

A measurement-based analysis is often challenged by incomplete data. Because the protocol consisted of hundreds of recordings per participant, it was likely that the equipment sometimes failed, which was the case with our recorded data. The voids were of two kinds: isolated random missing fluency values and the missing sequences that corresponded to one session (for jerk measurements). To handle the voids in recordings, we first compared the fitting accuracy for the (selected) participants' complete samples of data with all (sometimes incomplete) participants' sample data, to check for possible inconsistency. There were a total of 33 *incompletely* measured tracks of participants, for which we performed fitting of the exponential function, whereas there were a variable number of *full* training tracks evaluations, depending on the fluency score (GE: 27, JE: 20, IM: 27, and CT: 30). Obviously, we aimed at including the incomplete samples in the study, to increase the sample size and thus the reliability of the prediction.

**Figure 5:**
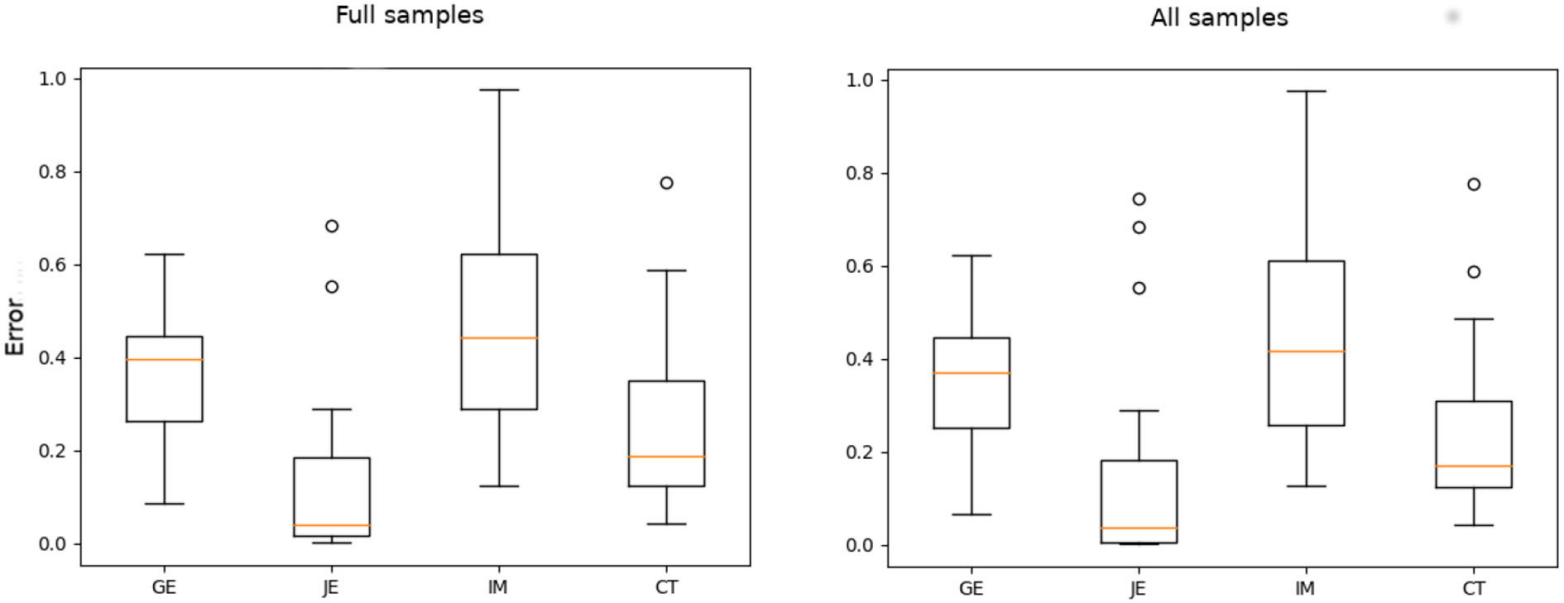
Fitting evaluation for full track data of the subset of samples **(left)** and the incomplete tracks of all the participants **(right)**. The figure indicates the median of mean squared error (MSE).

From Figure 5, we can observe that difference in fitting accuracy for incomplete and complete training tracks was in favor of full training tracks (with an exception of the time indicator CT, which, however, was not taken into account as a valid measure of fluency in our study, as mentioned in the beginning of the current section). For this reason, we may expect that, by imputing voids with appropriate data (e.g., using a missing data reconstruction machine learning algorithm), we could achieve a more accurate prediction. It is worth noting, however, that the two statistical tests, Mann–Whitney U-test and Kruskal–Wallis *H*-test (applied separately to each indicator), performed on the full track data and the data with voids, did not manifest statistically significant difference in the distributions of fitting results. For that reason and for the sake of simplicity (since the fitting accuracy difference was not substantial), we assumed in the following analysis that we could rely on curve fit to the corrupted signals (i.e., the data including missing values, without reconstructing the data).

In addition, in both cases (complete and incomplete datasets), three samples were eliminated due to the divergence problems of the fitting algorithm. The significantly divergent three samples of data to which the exponential function could not be fitted may have been the result of the different initial internal dynamics of the three participants, which had been established before training [we can suspect that there was a competition between the initial intrinsic dynamics and the task dynamics, which would explain the poor performance improvements observed in these participants, at least initially Kostrubiec et al. (2012)]. Due to the first result obtained, we were not reluctant to finally proceed with the 30 samples based on the incomplete datasets.

### 3.2. Predictivity evaluation

We aimed to examine the predictivity of the sets of each practicing group (VP1, VP2, and CP), by evaluating transfer test prediction stability (i.e., error variability).

We assessed the quality of the prediction with error *err*^*pr*^ defined in Algorithm 1 (line 20). The final evaluation to be depicted in Figures has been based on the median of the error vector that is the output of the Algorithm 1 (the result of the LOO procedure).

**Figure 6:**
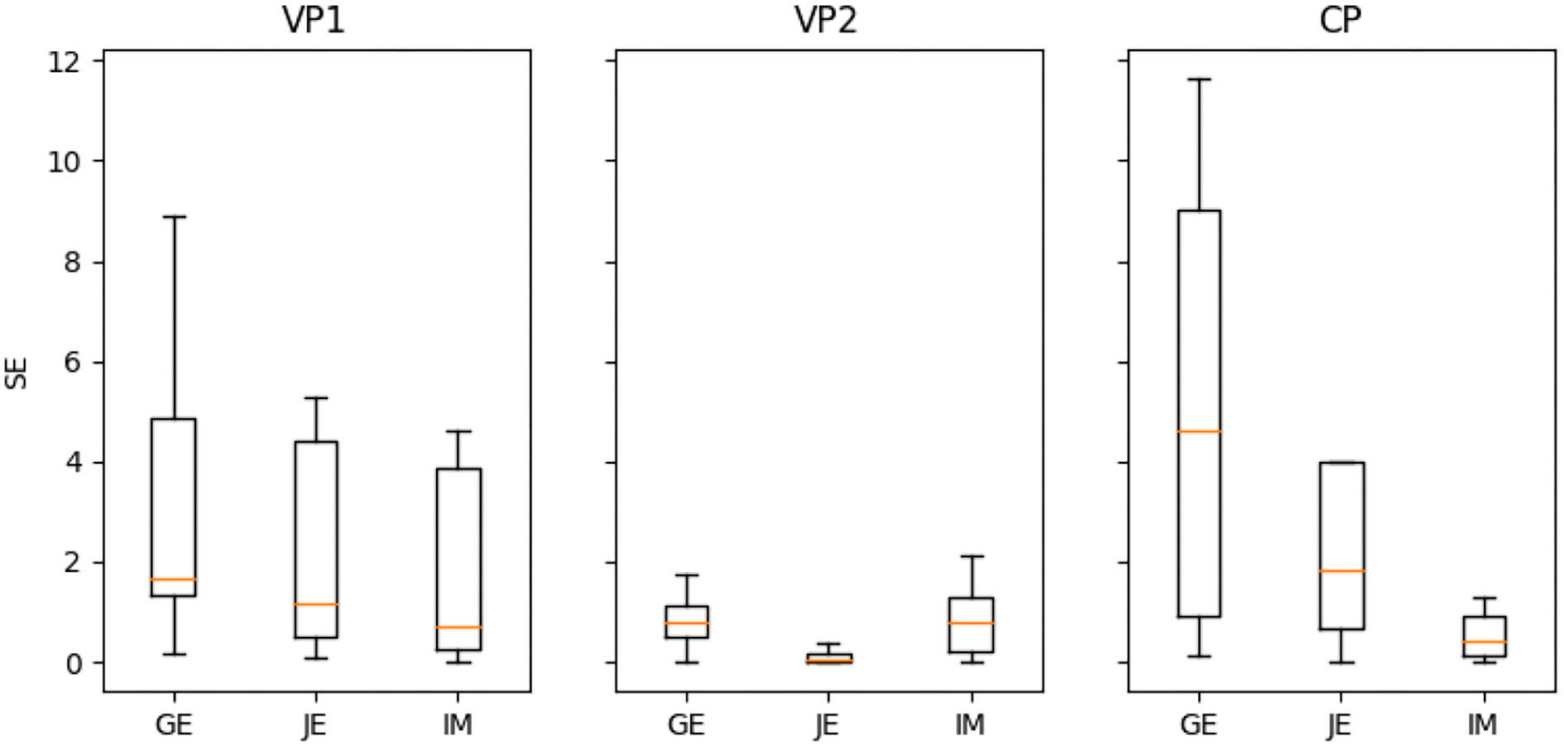
Comparison of the predictive power (due to square error SE) for variable practice (VP1 and VP2) and constant practice (CP) groups.

As Figure 6 demonstrates, the VP2 group excelled over the other two groups VP1 and CP by the measure of squared error (SE) variability in prediction (i.e., the adjustment of the prediction to the true value) and SE median (except for the immobility measure for CP). We recognize the lower variability of entropy and jerk metrics as an effect of more appropriate attribution of the prediction result to the learning set, which would be accurate in the case of the variable practice dataset VP2. The VP1 entropy SE is lower than CP entropy SE, but this pattern is reversed for a jerk (CP jerk SE is lower than VP1 jerk SE). The immobility metric demonstrates superior prediction for CP SE, whereas higher SE for both variable practice groups (and highest for VP1). However, it is worth noting that the algorithms that were utilized for the prediction are not designed for discrete feature values. Since the immobility ratio is based on the threshold for describing mobile and immobile action, the machine learning procedure is not guaranteed to be properly adjusted to this discrete score. The IM inverted effect could be a result of not meeting the assumption that the IM measure is continuous (it is based on an arbitrary threshold value, as defined in Supplementary Section 1), thereby demonstrating low prediction reliability. To support the hypothesis that IM differs in nature from the remaining (continuous) metrics, we may also verify the Lasso pre-train selection result (STEP 1 in Algorithm 1): We found that the following number of features was retained for the second step (refinement of the model): GE: 5, JE: 4, and IM: 1 (Table 2). In the case of IM, thus, only one feature was kept as important (providing very sparse result), which confirms the fact that this particular fluidity indicator is not very informative and the output assigned to input might be too elusive for Lasso algorithm to be properly predicted. Another reason for the misbehavior of the immobility ratio might be the sheer nature of this purely temporal indicator. In considering the nature of each one of the indicators, we might discuss the fluidity aspects that each of them prioritizes: whether fluidity should reflect efficient movement toward the end of the route (ultimate goal), or with general agility, allowing different ways of completing a given stretch of the route to be tested (exploration of multiple manners of goal reaching), even if, at the expense of efficient movement, it entails to move temporarily away from the goal. We can note that the rapid movements (that are given priority in the temporal fluency indicators) might impede the overall smoothness evolution in climbing. From our result, we conclude that our approach is appropriate mainly for measures that bear the characteristics of displacement (entropy and jerk) and not merely the temporal ones (immobility).

**Table 2:** Lasso feature selection result (*a*-additive term of fitting function = the maximum performance, *e* - exponential term of fitting function = the learning rate).

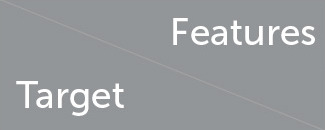	**GE**		**JE**		**IM**		**CT**	
	***a***	***e***	***a***	***e***	***a***	***e***	***a***	***e***
***y* ^*GE*^**	0	1	0	1	1	1	1	0
***y* ^*JE*^**	1	0	1	1	0	0	0	1
***y* ^*IM*^**	0	0	0	0	0	0	1	0

To validate the results with statistical tests, we used Kruskal–Wallis *H*-test (KW) (Kruskal and Wallis, 1952) and Mann–Whitney *U*-test (MW) (Mann and Whitney, 1947). These tests are recommended in case the compared sample sizes vary. The initially applied KW test (for VP1, VP2, and CP; df=2) demonstrated the only significant difference for the jerk score (*p* = 0.001 with *H* = 10.227), even though for entropy it was close to significance (*p* = 0.076, *H* = 3.157); immobility exhibited no difference (*p* = 0.776, *H* = 0.081). Furthermore, group pairwise MW test analysis revealed that only the entropy and jerk SE for the VP2 group vs. other groups VP1 and CP indicated significantly different distribution. The MW test values (*p*- and associated *U*-value) are illustrated in Table 3 (we did not find sufficient statistical significance in the distributions of our results for IM, as *p*>0.05). These findings further support the fact that the prediction stability that was significantly higher for VP2 in the case of GE and JE compared to the other groups (VP1 and CP) should be valid, in contrast to the opposite result obtained for the IM (which provided higher prediction stability for the CP group), and unlike the comparison between the VP1 and CP group (Figure 6).

**Table 3:** Statistical significance *p* (with *U*-value in brackets) for the sets with Mann–Whitney *U*-test (MW).

***p (U)***	**GE**	**JE**	**IM**
VP1 vs VP2	**0.041**(29)	**0.001** (9)	0.402(50)
VP1 vs CP	0.362 (36)	0.329 (35)	0.329 (35)
VP2 vs CP	**0.035** (28)	**0.001** (10)	0.322 (47)

### 3.3. Recap of the results

We have indicated the set of climbers VP2 as more predictive of transfer^3^, but additionally, by means of Lasso selection method, we revealed the fluency indicators (features being their parametric representation terms *a* or *e*) that could affect the prediction of skill transfer fluency. Therefore, we might suspect that spatial metrics are more adapted for this purpose, which may suggest their usefulness in future studies of dynamic variables that address the generalization property of the climber.

## 4. Discussion

In our analysis, we attempted to quantify the effect of three different practice conditions on the transfer evaluation stability (the magnitude of prediction error) with machine learning and its impact on attunement of the learner's dynamical variables to the most important information (Pacheco et al., 2019). In the discussion, we will comment on the methodological issues and state how, by solving them, we were able to make reference to the state of the art in motor learning science.

### 4.1. The methodological challenge

From the machine learning perspective, one important adversity in our study is the sample size, which is quite limited due to the complexity of data collection and ways of measuring it. In machine learning, a small sample size is a factor that strongly undermines the effectiveness of the methods used, reducing their reliability. For that reason, it was a noteworthy challenge to handle prediction with the large number of features, in comparison with a small number of samples in the datasets. We paid special attention to the correct selection of the final pipeline for the movement science framework application. Our solution to this problem was to 1) reduce long sequences of measurements to the parameters of the fitted curve and 2) introduce a pre-training step into the prediction algorithm. Nevertheless, we may notify that finding other ways of feature selection methods may result in other variants of the algorithm. Thus, further exploitation of other types of methods to reduce the complexity of the input data is highly desirable, given the importance of the ratio of the size of the input set to the number of parameters in statistical learning.

A similar issue is the heterogeneity of the sample, i.e., inter-individual variability among participants, and whether this has affected the generality of statistical methods in use. It is well-known that the averaging (the standardization or normalization of data required by particular algorithms) from motor learning perspective may intrude or falsify the individual dynamics of each participant (Newell and Liu, 2012). Although we may suspect that the algorithm accounts for the subtle structure of the data, we did not focus on it in our analysis by, for example, clustering the different types of learning dynamics among the participants; thus, this question remains open for future studies.

An important type of challenge in machine learning applied to behavioral signal analysis that should be highlighted is the need to handle incomplete data problems and the need for data imputation. The original signal (entropy, jerk, immobility, and climbing duration time sequences) to which we fitted the exponential function contained missing data, so finding an imputation method for an approximate value instead of ignoring the voids could impact the results of our prediction accuracy. In this view, research into an adequate approach to address the missing data problem (although not applied here, but inspired by Figure 5) in the case of a behavioral signal would be beneficial for improving the quality of the final prediction.

Once the limitations of the method are known and we are convinced that we have successfully addressed them in our approach, we are ready to discuss the results from a human movement science perspective.

### 4.2. Implications for human movement science

Induced variability in climbing tasks is straightforward to apply through handhold manipulation, as in our case. In our study, the task variants were designed by displacing the holds, that is, the modifications were applied to only one dimension during the practice sessions. The same dimension was manipulated to design the transfer test, to account for the same individual variables of the climbers' intrinsic dynamics. According to ecological dynamics (Button et al., 2020), the climber learns to continuously adapt to a set of interacting constraints (task, environment, personal resources) and attune himself to relevant opportunities for action. Therefore, ecological dynamics hypothesize that variable practice further increases adaptive behavior in the sense that climbers would learn to adapt more functionally (i.e., facilitating transfer and generalization). Attuning to variables that facilitate transfer to a new motor condition is considered a crucial part of the learning process, which in our case has taken place during training sessions. Then, the contextual change, that the learner faces in the transfer test trial, accounts for the adaptation of the learned variables within the reduced dimensionality, which supports transfer to new contexts in the case of a well-trained climber. Our results have pointed to the added value of the variable practice for skill acquisition and transfer and are consistent with Shea and Morgan's (1979) pioneering study demonstrating greater transfer when the three motor tasks were presented in a blocked vs. random sequence. Furthermore, our findings revealed that the positive effect of the variable practice was particularly significant when the variability of practice was self-controlled by the learner.

In our study, we revealed that in the self-controlled practice (VP2) the climber's intrinsic dynamics cooperate to provide more useful variables that guarantee stable performance in the transfer trial (Smeeton et al., 2013). In light of our results, the self-controlled practice learners were able to generalize more effectively in the sense that their intrinsic dynamics constituted during practice allowed for reduced randomness in learning curve adjustment and transfer fluency. These results might be explained by the fact that variable practice actively led climbers to successfully find more reliable information to tune to. However, the variable practice group (VP1) did not demonstrate improved predictions of their performance in the transfer test relative to the constant practice group. Overall, these results suggest that VP2 participants primarily benefited from a lower level of induced variability (compared to VP1) which gave them more opportunities to stabilize their discovered behavioral solutions practice (especially important for novices Ranganathan and Newell, 2013). Indeed, since VP2 could decide to practice on the same route for several sessions, they would make improved exploitation of route properties and optimize their chain of actions, whereas the rate of route changes in VP1 may have been too high for some participants. Thus, these results confirm and expand the previously acknowledged positive effects of self-controlled practice schedules on skill acquisition (Liu et al., 2012).

#### 4.2.1. Nature of fluency dynamics

One important aspect of our climbing experimental protocol is that climbers not only attempted to climb the route (to reach the last handhold on the trial route) but also to improve their fluency according to the feedback score from the previous session. This caveat made it possible to quantify the learning effect with fluency measures but at the same time emphasized the focus on the quality of the movements on the way to the goal, making it difficult to disentangle the effects of task and target on the learners' functional dynamics (Pacheco et al., 2019). However, we could still question to what extent the parameters of the exponential function of fluency history deployed in the study remain related to the subjects' intrinsic dynamics. Intuition would suggest that they are strongly associated with the parameters of the jerk score exponential fit, for which we obtained the lowest level of prediction error. Moreover, the jerk and entropy values were equally prominently represented among the selected features in the pre-training step of the machine learning algorithm. We can speculate that further research on how spatial fluidity corroborates particular aspects of dynamics during learning may elucidate the details (particular movement types) that allow contextual tuning of individual learners. Specifically, some research with various feature selection methods, that further structure (e.g., with feature grouping) the type of relevant indicator in use as an input (Tibshirani et al., 2005; Yuan and Lin, 2006; Jacob and Obozinski, 2009), may be helpful in understanding the particular contribution of fluency indicators (entropy, jerk, or immobility) to test score outcomes.

### 4.3. Concluding remarks

In summary, we have demonstrated that the self-controlled variability in practice induces greater stability in the individual's learning dynamics than in the absence of modification.

The methodology presented here allowed a statistical study of the behavioral signal with a small dataset; however, some methodological improvements could help to constitutionalize the insights obtained, and in this direction (e.g., solving the problem of missing data or the selection method evolution) we envision potential for further development of research in machine learning applied to the behavioral neuroscience of human movement.

## Data availability statement

The datasets presented in this article are not readily available because it is a property of ANR France. Requests to access the datasets should be directed to guillaume.hacques@univ-rouen.fr; ludovic.seifert@univ-rouen.fr.

## Ethics statement

Ethical review and approval was not required for the study on human participants in accordance with the local legislation and institutional requirements. The patients/participants provided their written informed consent to participate in this study. Written informed consent was obtained from the individuals for the publication of any potentially identifiable images or data included in this article.

## Author contributions

AA-S wrote the first draft of the manuscript and produced the main results as well as their statistical analysis. GH and LS contributed to the writing and revision of the first and last sections. RH and GG contributed to the writing and revision of the second and third sections of the manuscript. GH provided the dataset and made the pre-calculation. All authors contributed to the conception and design of the study, read, and approved the submitted version.

## Footnotes

^1^Feedback was intended to provide participants with information about their climbing performance and guide their learning. The aim was to encourage participants to discover new ways of climbing the route and to fluently chain movements to lower their fluency scores to the greatest extent possible, without explicitly indicating how to do it (encouraging external focus of attention Wulf and Shea, 2002). Before starting a new session, the feedback from the previous session was described and explained to the participants. It was presented in the form of images of harness light trajectories on climbing routes during the session (one image per climb, as in Supplementary Figure S3, Supplementary Section 2.1) and the corresponding values of three fluency scores labeled spatio-temporal fluency (JE), spatial fluency (GE), and immobility (IM). They were informed that the drawn line corresponded to the trajectory and that the more direct it is, the lower the spatial fluency score (GE) would be. The temporal fluency score (IM) was described as the percentage of climbing time spent immobile and the spatio-temporal score (JE) as the measure of saccadic movements during climbing (“knots” on the trajectory line). The participants were also informed that their aim was to reduce these scores to the greatest extend during training sessions.

^2^Participants were not instructed to climb fast, but as *smoothly* as they could.

^3^The results for variable practice VP=VP1+VP2 vs. control practice CP can be found in Supplementary Section 2.
