# Supplementary material for: Evaluating transfer prediction using machine learning for skill acquisition study under various practice conditions
Source: Front Psychol. 2023 Jan 19;13:961435. doi: 10.3389/fpsyg.2022.961435 (PMC9937057; doi:10.3389/fpsyg.2022.961435)
Supplement: Supplementary file 1 [file Data_Sheet_1.PDF]

frontiers

Supplementary Material

1 SUPPLEMENTARY DEFINITIONS

Here we add the definitions of precalculated fluency metrics.

1.0.1 Geometric index of entropy

Due to (Cordier et al., 1994; Orth et al., 2017)

$$GE = \frac{1}{log(2)} log\Big(\frac{2 \cdot l}{c_l}\Big),$$

where $l = \sum_{i=1}^{n} \sqrt{(x_i)^2 + (y_i)^2}$ is length of the climbing trajectory and c_l is a perimeter of the trajectory convex hull (its unit is [bit] or [log2]).

1.0.2 Jerk of hip acceleration

Jerk is a spatio-temporal measure, that can be calculated either as third time derivative of position or the rate of change of acceleration.

As in (Seifert et al., 2014)

$$JE_s(T) = C \int_0^T ||x_s||^2 ds,$$

where $x_s : [0, T]$ is a smooth trajectory developed in time (s), $C = \frac{T^5}{(\Delta x)^2}$ is a normalization constant, and T is duration of climbing. Herewith defined jerk is a dimensionless measure.

1.0.3 Immobility ratio

From (Orth et al., 2017), for a trajectory $X : [O, T] \longrightarrow \mathbb{R}^2$, we find the threshold based immobility to mobility ratio (with unit in [s]) as:

$$IM(X) = \frac{\sum_{i=1}^{n} p_i}{n},$$

$$p_i = \begin{cases} 1 & \text{for} \quad v_i < threshold, \\ 0 & \text{for} \quad v_i \geq threshold, \end{cases}$$

$$v_i = t_i \sqrt{(x_i)^2 + (y_i)^2} = t_i l_i,$$

where $i = \{1, ..., n\}$ (n is the number of time intervals) and (x_i, y_i) symbolizes the coordinates of the body centre position in two-dimensional space and l_i is its temporal displacement (a part of the trajectory). In our analysis $threshold$ velocity is $20cm/s$.

2 SUPPLEMENTARY RESULTS

The results provided here concern the variable practice group (VP that consists of VP1+VP2, as explained in the main article) comparison to control practice group (CP)[1].

The Fig. S1 reveals the overall lower squared error (SE) variability, as well as SE median in case of the VP data set. We recognize the lower variability of the entropy and jerk metrics as an effect of a more appropriate assignment of the prediction score to the learning set, which would be more accurate in case of variable practice VP (VP1+VP2) data set. In order to find the statistical significance of the results obtained (the prediction stability of the transfer outcome value for all the indicators GE, JE and IM) between the two sets (variable and constant practice group), we applied two statistical tests: Mann-Whitney U Test and Kruskal-Wallis H Test (Kruskal and Wallis, 1952; Mann and Whitney, 1947). They revealed the following values respectively: for entropy MW: $p = 0.015$ and KW: $p = 0.028$; for jerk MW: $p = 0.002$ and KW: $p = 0.003$, for immobility MW: $p = 0.41$ and KW: $p = 0.803$, thus, as previously in a VP subgroup case, we did not find sufficient statistical significance in the distributions of our results for IM ($p > 0.05$). These findings further support the fact that the prediction stability that was much higher for VP in case of GE and JE should be valid, unlike the contrary result obtained for the IM (higher prediction stability for CP group), Fig.S1.

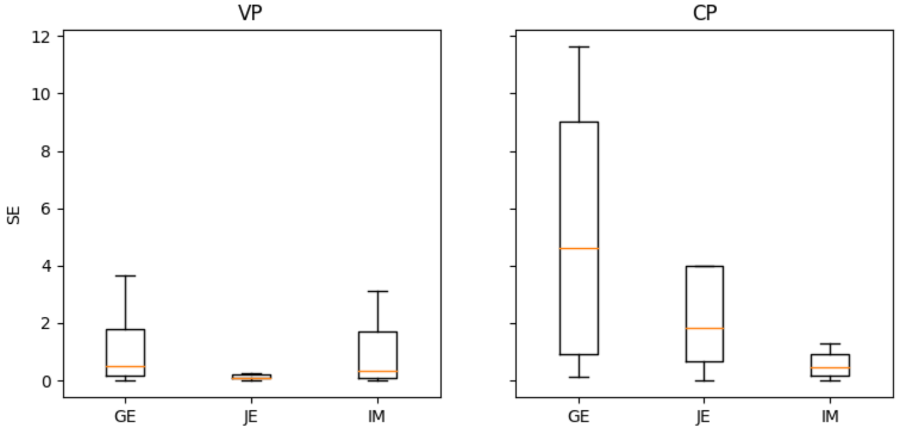

Figure S1. Comparison of the predictive power (due to squared error SE) for variable practice (VP = VP1+VP2) and constant practice (CP) groups.

2.1 Supplementary Figures

2.1.1 Route design

Fig. S2

2.1.2 Feedback example

Fig. S3

[1] It should be noted, that in this case the balance between the groups is affected, as there were 21 participants in VP versus 9 participants in CP.

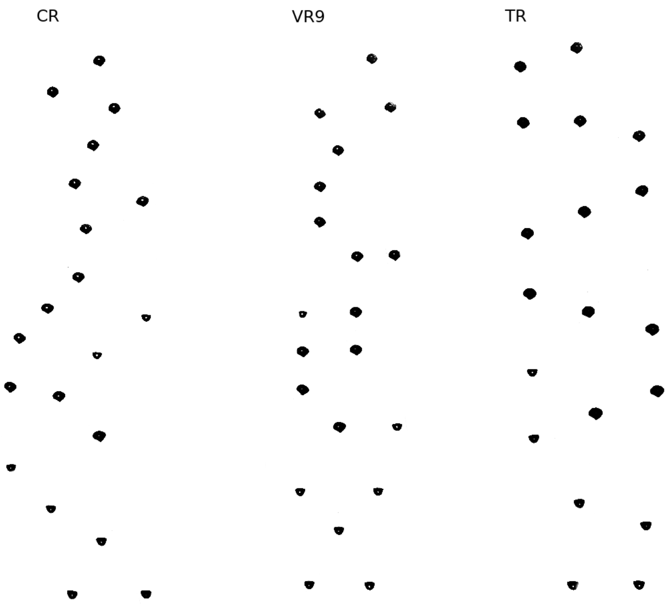

Figure S2. The examples of routes climbed in the experiment. Left: constant route, middle: one of the variant routes (last variant applied during practice), right: transfer route.

REFERENCES

Cordier, P., France, M., Pailhous, J., and Bolon, P. (1994). Entropy as a global variable of the learning process. *Human Movement Science* 13(6), 745–763

Kruskal, W. and Wallis, W. (1952). Use of ranks in one-criterion variance analysis. *Journal of the American Statistical Association* 47, 583–621. doi:10.1080/01621459.1952.10483441

Mann, H. and Whitney, D. (1947). On a test of whether one of two random variables is stochastically larger than the other. *The annals of mathematical statistics* , 50–60

Orth, D., Kerr, G., Davids, K., and Seifert, L. (2017). Analysis of relations between spatiotemporal movement regulation and performance of discrete actions reveals functionality in skilled climbing. *Frontiers in Psychology* 8, 1744. doi:10.3389/fpsyg.2017.01744

Seifert, L., Orth, D., Boulanger, J., Dovgalecs, V., Hérault, R., and Davids, K. (2014). Climbing skill and complexity of climbing wall design: Assessment of jerk as a novel indicator of performance fluency. *Journal of Applied Biomechanics* 50(5), 619–625

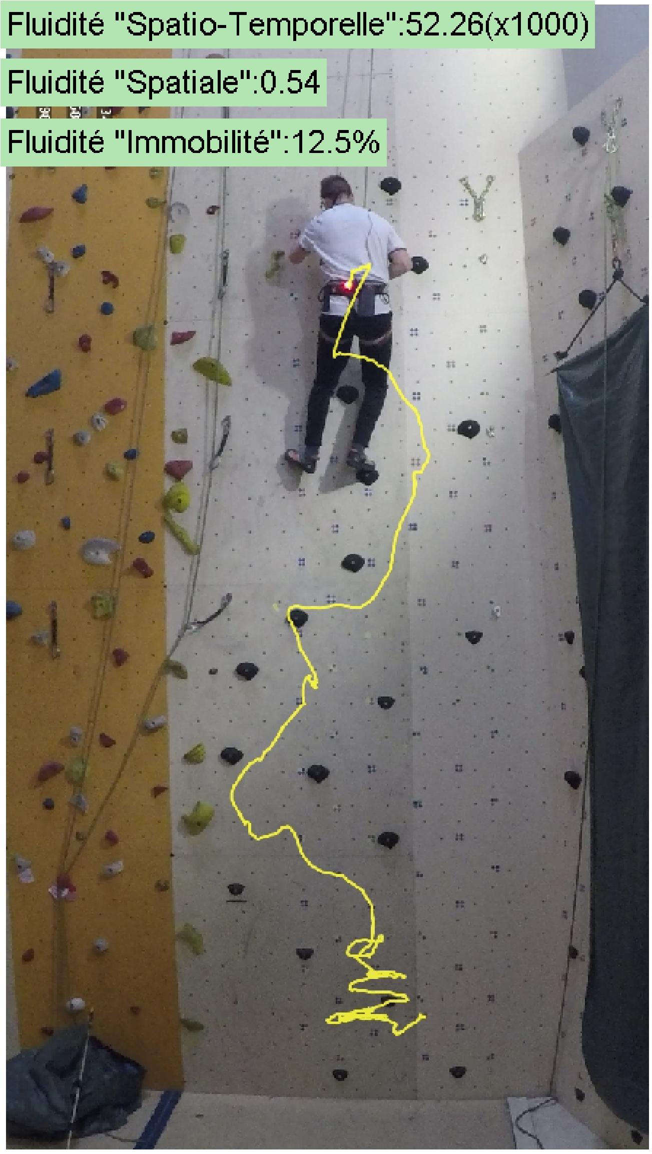

Figure S3. The example of feedback image with the trajectory (yellow line) and metrics of one climbed route given to participant.
